# Heat Stress Nephropathy in CKD of Uncertain Etiology Hotspots of Bargarh District Odisha, India

**DOI:** 10.1016/j.ekir.2025.07.013

**Published:** 2025-07-15

**Authors:** Pralaya Biswas, Ashish Kumar Sahu, Sourav Shristi, Tapan Kumar Behera, Sawan Kumar Sahoo, Syed Nikhat Ahmed, Sarat Kumar Mohanty, Sharada Shrinivas Pati, Kailasam Murugesan, Niranjan Mallick, Pradeep Kumar Naik, Sunanda Nayak, Iswar Baitharu

**Affiliations:** 1 Renal Toxicopathology and Medicine Laboratory, P.G. Department of Environmental Sciences, Sambalpur University, Sambalpur, Odisha, India; 2 Biochemistry Laboratory, School of Life Sciences, Sambalpur University, Sambalpur, Odisha, India; 3 Department of Biotechnology and Bioinformatics, Sambalpur University, Sambalpur, Odisha, India; 4 Research and Development Wings, State Pollution Control Board, Bhubaneswar, Odisha, India; 5 Department of Nephrology, Veer Surendra Sai Institute of Medical Science and Research, Burla, Odisha, India; 6 Department of Pathology, Veer Surendra Sai Institute of Medical Science and Research, Burla, Odisha, India

**Keywords:** Bargarh, CKDu, dehydration, farmers, heat stress nephropathy

## Abstract

**Introduction:**

Repeated heat exposure, physical exertion, and inadequate hydration can cause acute kidney injury (AKI), potentially progressing to chronic kidney disease (CKD). However, cohort-level research on heat stress as a contributing factor remains limited. This study investigates occupational heat exposure among farming communities in hotspot villages of Bargarh district, Odisha, India.

**Methods:**

A cross-sectional study of 1136 participants was conducted in Bargarh district to assess heat stress nephropathy among agricultural workers. Based on Sri Lankan criteria, heat stress nephropathy was defined by albumin-creatinine ratio ≥ 30 mg/g, no known CKD causes, urine specific gravity ≥ 1.03, and signs of tubulointerstitial nephritis. Heat stress index, serological tests, and urine analyses were performed using standard protocols. Serum and urine levels of heat shock protein (HSP)27 and HSP70 were measured via enzyme-linked immunosorbent assay. Renal biopsies were conducted on 8 selected patients for histopathological evaluation.

**Results:**

Out of the total screened population, 157 potential cases of heat stress nephropathy were identified, with 63.3% of affected individuals being farmers. The Attabira block recorded the highest heat stress index and the most CKD of uncertain etiology (CKDu) cases among farmers. Markers of dehydration, including the simplified wet bulb globe temperature (sWBGT) index, urine specific gravity (64.09%), albumin-to-creatinine ratio (5.88%), blood urea nitrogen (BUN, 63.2%), and HSP, were significantly elevated in the farming population compared with the control group. Renal histopathological analysis revealed tubulointerstitial nephritis with signs of fibrosis.

**Conclusion:**

Heat stress nephropathy was commonly observed among individuals involved in farming activities. Renal histopathological analysis, along with elevated levels of HSP70 and HSP27 confirmed the diagnosis.

## Graphical abstract

**Figure:**
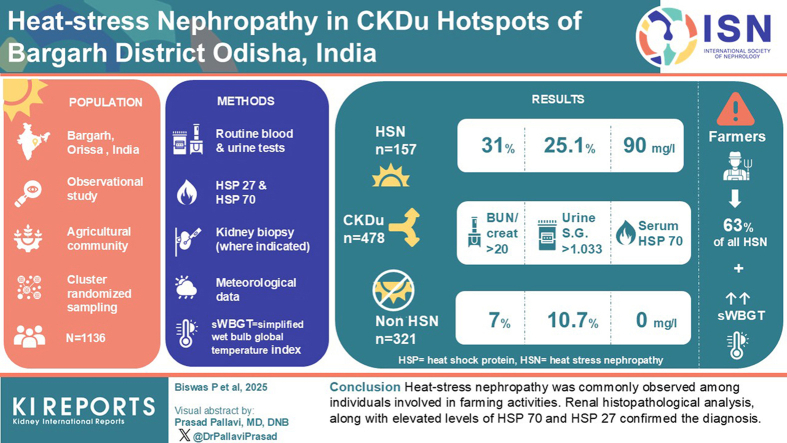

Heat stress is increasingly recognized as a key contributor to CKDu, particularly in tropical regions with consistently high temperatures.^1^ Repeated exposure to heat, combined with strenuous physical labor and inadequate hydration, can cause recurrent AKI that progresses to CKD, commonly termed heat stress nephropathy.^2^ This mechanism is widely accepted as the primary driver of CKDu in Central America. In India, many high-burden CKD regions are home to laborers working under hot, humid conditions, making them especially vulnerable. Rising global temperatures and limited access to clean drinking water may exacerbate the risk of recurrent dehydration and kidney damage in both outdoor and indoor workers. These conditions underscore the urgent need for identifying occupational risk factors and implementing effective preventive strategies. The close link between high environmental temperatures and CKDu has led to the proposal of the heat stress/dehydration hypothesis as a plausible explanation for heat stress nephropathy, also referred to as global warming nephropathy—the first epidemic attributed to climate change.^3^ Epidemiological studies from Iran, Ethiopia, Sri Lanka, Uddanam (India), and Nicaragua have reported a 10% to 20% prevalence of heat stress nephropathy among agricultural workers. In Central America, particularly among sugarcane workers, this condition is now widely known as Mesoamerican nephropathy,^4^ with similar patterns observed in Sri Lanka’s farming communities.^5^

Repeated heat stress and cyclic dehydration can lead to hyperosmolarity, vasopressin activation, increased urinary concentration, and renal vasoconstriction, resulting in subclinical ischemic kidney injury that may progress to CKD.^6^ Kidney biopsies from affected individuals in Mesoamerica reveal tubular injury and focal fibrosis.^7^ Dehydration-induced hypovolemia can cause prerenal AKI, which is potentially reversible with timely rehydration.^8^ Elevated plasma osmolarity also activates aldose reductase in the proximal tubule, promoting endogenous fructose production; its metabolism via fructokinase triggers inflammation and renal damage.^9^ The consumption of sugary beverages instead of water—commonly observed among Mesoamerican sugarcane workers—further exacerbates this injury.^10^ In addition, volume depletion can lead to hypokalemia, intrarenal vasoconstriction, and hypoxia, resulting in tubulointerstitial damage.^11^ Strenuous labor under heat stress may also cause subclinical or clinical rhabdomyolysis, a known risk factor for recurrent AKI and subsequent CKD.^12^ Dehydration contributes to hyperuricemia and uricosuria, promoting crystalluria, which has been documented in sugarcane workers.^13^

Heat exposure was assessed using the sWBGT, which accounts for temperature, humidity, wind speed, sun angle, and cloud cover, and is a standard measure of heat stress in direct sunlight.^14^ A heat stress index between 41 °C and 54 °C is classified as dangerous and can lead to heat cramps, heat stroke, and severe dehydration. Under such conditions, the heat shock response is triggered by factors such as reactive oxygen species and elevated urea levels.^15^ Whereas some HSPs function as molecular chaperones under normal conditions, others are induced during stress to maintain protein homeostasis and inhibit apoptosis. HSP27, a small HSP, prevents apoptosis by blocking caspase activation and possesses antioxidant properties that support redox balance.^16^ HSP70 inhibits both intrinsic and extrinsic apoptotic pathways^17^ and has been shown *in vitro* to protect against urea-induced cellular damage. During CKD progression, cellular injury may lead to the extracellular release of HSPs.^18^ Accordingly, we measured serum and urine concentrations of HSP27 and HSP70 in the study population.

Following the commissioning of the Hirakud Dam, Bargarh district in western Odisha experienced uninterrupted irrigation, transforming it into an agricultural hub, particularly for paddy rice cultivation. This intensification, however, has led to increased use of agrochemicals and pesticides, which have been associated with increasing community health issues, including cancer and CKD. Previous studies from our laboratory have implicated pesticides and heavy metals in the onset and progression of CKD in the agriculturally intensive zones of Bargarh.^14^ In addition to agrochemical exposure, farmers in this region are regularly subjected to extreme environmental heat during work hours. Bargarh shares agroclimatic features with Uddanam in Andhra Pradesh—both regions experience tropical climates, temperatures exceeding 40 °C, high humidity, marked seasonal variation, and intensive farming. This study investigates the potential link between heat stress exposure and nephropathy among farmers in Bargarh, representing the first comprehensive assessment of heat stress nephropathy in the region, including measurement of HSP levels in affected individuals.

## Method

### Study Site

**Figure 1:**
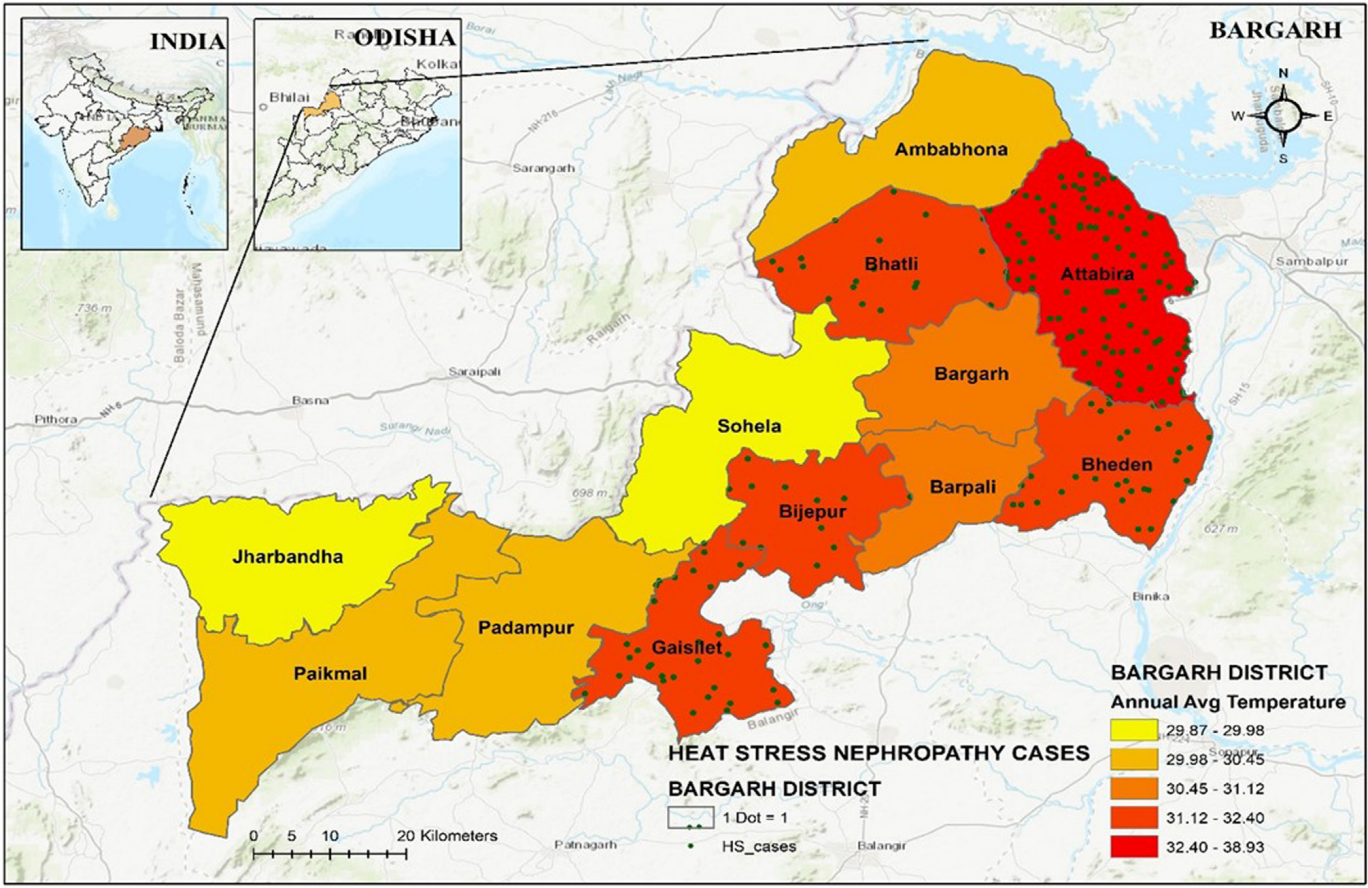
Map showing annual average temperature and incidence of heat stress nephropathy cases in studied blocks of Bargarh district, Odisha, India.

Bargarh district once regarded as the rice bowl of the state, is situated at the western part of Odisha, India. The district comprises a total of 12 blocks. It is geographically located at 21.33° N, 83.62° E, having a total population of 1,481,255 out of which males and females are 749,161 and 732,094, respectively (census report 2011). A current study from our laboratory has identified 16 CKDu hotspot villages that are under 5 blocks, namely Bijepur, Gaisilate, Bhatli, Bheden, and Attabira at Bargarh district.^19^ All these blocks fall under an intense agricultural zone. Majority of the population practice agricultural activities in the district, with paddy rice as a major crop grown in this area. Approximately 60% of the cultivated land in the district is irrigated under the Hirakud command area. The mean annual temperature of these hotspot areas is > 50 °C and the area has high relative humidity (Figure 1).

### Research Design and Study Population

A cross-sectional community-based survey was conducted in 2024 to assess the prevalence of CKD in 5 identified CKD hotspot blocks of Bargarh district using clustered random sampling and probability proportionate to size methodology. The population was divided into clusters, where cluster villages were heterogeneous with respect to socioeconomic status, occupation, age, level of education, and all the clusters selected fall under an intensive agricultural zone that shares homogeneity between the clusters. All the clusters were assigned a unique identifier followed by a lottery to ensue randomization. To determine the sample size from each cluster at first sampling, factor (f) was determined using the following formula:^20^

$$f=n\;/\;N_{\text{total}}$$

Where, f = sampling factor

*n* = total sample size

*N*_total_ = total population of each cluster/block

Then the sample size for each cluster (*n*_i_) was determined using the following formula

$$n_{i}=N_{i}\;x\;f$$

Where, *n*_i_ = sample size of each cluster

*N*_i_ = population of each cluster

f = sampling factor

**Figure 2:**
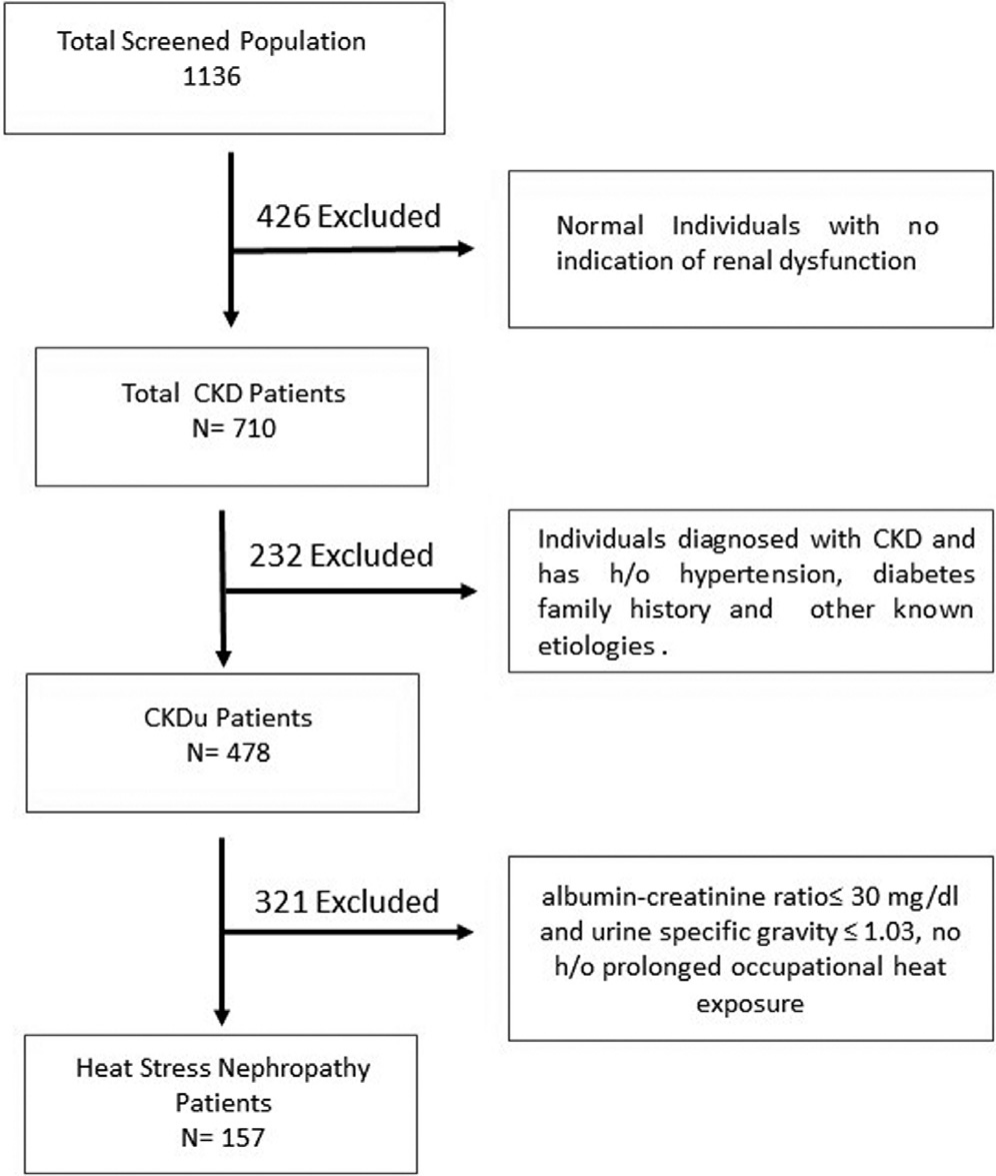
Consort flow diagram of the screened population. CKD, chronic kidney disease; CKDu, CKD of uncertain etiology.

Out of 1136 individuals screened, 656 were males and 480 were females. The study included participants aged ≥ 18 years who met the following inclusion criteria: voluntary participation with written informed consent and, for farmers, a minimum of 6 months of exposure to agricultural work. The exclusion criteria included refusal to participate, presence of non-CKD conditions affecting hematological profiles (e.g., hematological disorders, inflammatory conditions, cancer, and hemorrhagic episodes), and pregnancy or lactation. A CONSORT flow diagram (Figure 2) illustrates the screening process. Participants were categorized occupationally as farmers (working 7–8 hours daily in open fields, exposed to heat and pesticides), laborers (construction workers exposed to heat for 4–5 hours daily, minimal chemical exposure), and others (e.g., government employees, teachers, homemakers, and industrial workers, with limited heat exposure of 1–2 hours daily in well-ventilated environments). Ethical approval was obtained from the Institutional Ethics Committee of Sambalpur University (17/IEC-SU/2023, dated July 29, 2023), and administrative permission was granted by the district administration.

### Diagnostic Criteria and Definitions

CKD was diagnosed based on the Kidney Disease: Improving Global Outcomes guidelines as follows: estimated glomerular filtration rate (eGFR) < 60 ml/min per 1.73 m^2^ for ≥ 3 months and albuminuria or proteinuria (albumin ≥ 30 mg/g or protein-to-creatinine ratio ≥ 30 mg/g). Hypertension was identified by history (on treatment) or through screening, defined as systolic BP ≥ 140 mm Hg and/or diastolic BP ≥ 90 mm Hg on 3 separate occasions. Diabetes was diagnosed either by history or random plasma glucose ≥ 150 mg/dl. Patients with a history of prolonged hypertension, diabetes, or other known causes were classified as CKD with known etiology. CKD cases lacking such histories, or with only mild hypertension, protein creatinine ratio ≤ 1, progressive renal failure, and confined to specific geographical areas were categorized as CKDu. CKDu predominantly affected younger, low-income males working in hot climates with frequent dehydration. Heat stress nephropathy, as defined in Sri Lankan studies, included patients with albumin-to-creatinine ratio ≥ 30 mg/g, no known CKD causes, urine specific gravity ≥ 1.03, and features of tubulointerstitial nephritis.^5^

### Data Collection and Clinical Measurements

A standardized questionnaire, approved by the nephrologist at Veer Surendra Sai Medical College and Hospital and the Institutional Ethics Committee of Sambalpur University, was used to collect data on demographics (age, education, income, occupation), lifestyle (alcohol and tobacco use, daily water and fructose intake), health status (history of diabetes, hypertension, nonsteroidal antiinflammatory drug use, and other illnesses), and work history (workload, sunlight exposure, pesticide use). The questionnaire was translated into Odia (Supplementary Table S1), with Koshli/Sambalpuri language support provided to address literacy barriers. Each questionnaire took approximately 5 to 7 minutes to complete. Clinical measurements included blood pressure (average of 3 readings taken after 5 minutes rest using a validated digital monitor), height and weight (using a SECA stadiometer and OMRON digital scale) to calculate body mass index, and eGFR using the CKD-EPI equation.^21^ CKD progression was assessed through estimation of CKD stages based on eGFR values.

### Collection of Biological Sample and Analysis

#### Serological and Hematological Analysis

All analytical-grade chemical reagents were procured from Sigma Aldrich, unless stated otherwise. Blood samples were collected by a trained phlebotomist from peripheral veins using 3 types of vacutainer tubes, namely plain, ethylenediamine tetraacetic acid, and fluoride tubes. Samples were used to analyze serological parameters, hemoglobin, complete blood count, BUN, BUN-to-serum creatinine (SCr) ratio, serum uric acid (S-UA), and random blood sugar. Both the first morning void (5 ml) and 24-hour urine samples were collected in polypropylene containers. All samples were transported in ice boxes to the Regional Diagnostic Centre at Veer Surendra Sai Institute of Medical Science and Research, Burla, Odisha, and analyzed on the same day. Serum was separated by centrifugation at 3000 rpm for 10 minutes at 4 °C. Serological parameters and random blood sugar were measured using the Transasia XL 1000 Auto Analyzer (Germany), whereas hemoglobin and complete blood count were assessed via photometric method using Mindray BC 1000 6 Part (Germany). Urine protein was estimated using heat coagulation and corrected for creatinine. Urine creatinine was measured using the Jaffe’s kinetic method; urine REME tests were conducted using dipstick methods.

#### Assessment of HSP 27 and HSP 70 Using Enzyme-Linked Immunosorbent Assay Kit

Separate serum and urine aliquots were prepared from collected samples to quantify HSP27 and HSP70, which are established biomarkers commonly used to assess heat stress exposure. Enzyme-linked immunosorbent assay kits (DuoSet IC, R&D Systems, Minneapolis, MN) were used according to the manufacturer’s instructions. The color reaction was initiated with tetramethylbenzidine (Sigma, St. Louis, MO) and stopped with an acid solution, after which optical density was measured at 450 nm using a microplate reader. To account for fluctuations in urine concentration, fractional HSP excretion was calculated using the formula:

([urineHSP×SCr]/[serumHSP×urinecreatinine])×100.

#### Ultrasonography of Kidney and Urinary Bladder

Ultrasonography of kidney and urinary bladder was conducted to check for the presence of nephrolithiasis and size of the kidney. Ultrasonography of the kidneys and urinary bladder was conducted for all participants at Department of Radiology, VIMSAR, Burla using a high-resolution real-time ultrasound machine equipped with a curvilinear transducer (3.5–5 MHz) and a linear probe (7–12 MHz), where necessary.

#### Histopathological Study of Selected Patients With CKD

Patients who met the criteria for a kidney biopsy were carefully examined by a nephrologist at Veer Surendra Sai Institute of Medical Science and Research, Burla. These criteria included high SCr levels, kidney size of at least 9 cm on ultrasound, presence of hematuria, or significant proteinuria (> 1 g/d). Patients aged < 30 years with symptoms similar to CKDu, or those with kidney cysts, masses, or significant asymmetry in kidney size were also included. A total of 8 patients underwent biopsy following the advice of the nephrologist. The biopsy procedure was meticulously performed by a trained nephrologist guided by ultrasonography for precision. During the biopsy, renal tissue samples were carefully collected into screw-capped containers prefilled with 10% formaldehyde solution buffered to pH 7. These containers were immediately labelled with detailed patient identification to ensure proper tracking and handling. After collection, these labelled samples were transported to the histopathology section, Department of Pathology at VIMSAR, Burla. The samples were processed as per standard protocols, which included meticulous paraffin embedding of the tissue samples followed by precise section cutting. After sectioning, the sections were stained using eosin and hematoxylin, followed by histopathological evaluation using light microscopy. This meticulous process allowed for detailed histopathological evaluation aimed at obtaining a comprehensive diagnosis.

### Assessment of Heat Exposure

Temperature fluctuations across different parts of Bargarh district were assessed using rainfall, temperature, and humidity data collected from the Indian Meteorological Department, Bhubaneswar, Odisha, for the period 2015 to 2023. The extended timeframe of temperature data in this study was chosen to evaluate long-term environmental trends and occupational exposure patterns among the studied population, as well as their association with CKDu onset and progression, given that CKDu is a chronic disease with slow progression. Measurements were taken under typical field conditions, with sensors positioned at a height of about 1.5 m. The conditions during data collection included temperature range, humidity, wind speed, cloud cover, and seasonal variation. sWBGT was calculated as recommended by Dunne *et al.*^22^ as a proxy of heat exposure (cumulative effect of temperature and humidity) of occupational groups in their work environment. The formula used to determine sWBGT was:

sWBGT=0.7×Tw+0.3×Ta

where, Tw represents the isobaric wet bulb temperature and Ta denotes the dry air temperature at 2 m above ground level. sWBGT was measured at a continuous real time frequency. Subsequently, sWBGT values were computed using MATLAB (MathWorks Inc., USA) by inputting the Tw and Ta data into the equation. For analysis, sWBGT data collected between 0600 and 1800 hours were averaged to estimate the daily heat exposure experienced by the target communities during their work activities. The criteria for classifying heat stress were based on sWBGT index and level of physical exertion.

### Statistical Analysis

All the data were processed and analyzed using Microsoft Excel 2016 and IBM SPSS (version 25.0). Data were presented as mean ± SD. Difference between occupations were assessed using analysis of variance and Kruskal-Wallis tests for normally and not normally distributed continuous variables, respectively; and Spearman’s rank correlation test for categorical variables or Fisher’s exact test specially when chi-square test was not applicable. *Post hoc* test was applied to determine the associations between clinical and other parameters among the 3 studied groups. Level of significance during all the statistical test was set at *P* ≤ 0.05. Seaborn, a Python-based data visualization library and graphical tool was used to illustrate the relationship between 2 continuous variables. The link between the incidence of heat stress nephropathy and the heat stress index was explored using MATPLOTLIB package.

## Results

### CKDu is Most Prevalent Among Farmers

A total of 1136 individuals were screened in the hotspot villages using the cluster random sampling method. Among them, 710 individuals were found to be affected with CKD. Out of the 710 individuals, 478 patients were diagnosed with CKDu whereas the rest were found to be affected with CKD of known etiologies. Occupational stratification revealed that CKDu was most prevalent among farmers, with 57.5% of affected individuals belonging to this group. In addition, approximately 73% of CKDu cases were males, the majority of whom were engaged in farming.

### Socioeconomic Status, Intake of Sugary Beverage, and Lifestyle Varied Significantly Among the 3 Different Occupational Groups

The disease was more common among individuals with lower income and lower educational levels. A statistically significant difference was observed among the 3 occupational groups concerning heat stress index, daily heat exposure, agrochemical exposure, and intake of sugary beverages. Direct agrochemical exposure was highest among farmers, followed by laborers. Tobacco consumption was more prevalent among farmers, whereas alcohol consumption was highest among laborers. The mean age was highest among laborers (58.21 ± 9.31 years) compared with farmers (47.91 ± 10.02 years) and others (51.78 ± 11.10 years). A significant difference (*P* < 0.001) was found in gender distribution, with males predominantly represented among farmers compared with laborers and others. The level of education varied significantly, with illiteracy being highest among farmers and laborers, whereas all individuals in the “other” category had at least a primary education. Degree-level education was more common among individuals in the “other” category. Income distribution showed no significant differences across groups (Supplementary Table S2).

### Farmers Were Exposed to Prolonged Heat Stress and Agrochemicals Occupationally

Farmers experienced the highest heat exposure (6 ± 1 h/d) compared with laborers (4 ± 0.5 h/d) and others (2 ± 1 h/d), with a highly significant difference (*P* < 0.001). Similarly, the heat stress index was notably higher among farmers (118.96 ± 0.31) than among laborers (64.71 ± 0.28) and others (22.90 ± 2.66). Agrochemical exposure was most prevalent among farmers, minimal among laborers, whereas the “other” group had no direct occupational exposure. Water intake and sugary beverage consumption did not significantly differ across groups. A history of tobacco consumption was most common among farmers, followed by laborers and others, though the difference was not significant. However, alcohol consumption was significantly higher among laborers than among farmers and others. The prolonged use of nonsteroidal antiinflammatory drugs (> 3 months) was reported more frequently among farmers than among laborers and others, but the difference was not statistically significant (Supplementary Table S2).

### Several Biochemical Parameters Varied Significantly Among the Group

The prevalence of kidney stones was highest among laborers, whereas it was much lower among farmers and absent in the “other” category, though the difference was not statistically significant (Supplementary Figure S1). The history of urinary tract infections was highest in farmers (57.76%), followed by laborers (9%) and others (7.2%). Dysuria prevalence showed no significant difference across groups. Triglyceride levels varied significantly across groups, with farmers showing the highest levels compared with laborers and others (*P* < 0.001). Hematocrit levels were significantly different across groups, with the highest levels in farmers and the lowest in others. Similarly, hemoglobin levels were significantly lower in the “other” group than in farmers and laborers. White blood cell counts and neutrophil percentages showed no significant variation among different occupation group. Lymphocyte percentages were slightly lower in the “other” group than in farmers and laborers, but the difference was not statistically significant (Supplementary Table S3).

### Markers of Kidney Function Showed Variations Among Individuals of Different Occupational Groups

Farmers exhibited significantly higher BUN levels than laborers and the “other” group, with a greater proportion of farmers (15.8%) having BUN > 20 mg/dl (*P* = 0.015). Although SCr levels did not significantly differ between groups, elevated SCr (> 1.2 mg/dl) was more prevalent among farmers (19.2%). The eGFR was lowest among the “other” group, but significantly more of the farmers (54.81%) had eGFR < 60 ml/min per 1.73 m^2^ compared with laborers (22.05%) and the “other” group (8.82%) (*P* < 0.001). S-UA levels showed no significant differences, but farmers had the highest percentage of elevated S-UA (> 7.2 mg/dl). Proteinuria (> 30 mg/d) was most frequently observed among farmers (252 cases), followed by laborers (50 cases) and the “other” group (9 cases). Blood in urine was most common among farmers (24.54%). Urinary specific gravity (U-SG) ≥ 1.0330 was also highest in farmers (45.30%), whereas urinary pH ≤ 5.5 was significantly more frequent in farmers than in other groups (*P* = 0.012). BUN-to-SCr ratio > 20 was significantly elevated in farmers compared to laborers and the “other” group. HSP levels varied significantly, with serum HSP27 and urinary HSP27 levels highest among farmers. Serum HSP70 was detected only in farmers, whereas urinary HSP70 levels were significantly elevated in farmers compared with other groups. The fractional excretion of HSP70 was also significantly higher in farmers than in laborers and the “other” group (*P* < 0.001) (Supplementary Table S4).

### Major Fraction of Farmers Suffering From CKDu Showed Symptoms Similar to Heat Stress Nephropathy

A total of 157 cases, possibly of heat stress nephropathy, out of 478 patients with CKDu were identified. Among the 3 occupational groups, farmers were the most affected, accounting for 63.6% of cases, followed by laborers (20.38%) and individuals in other occupations (15.92%). A significant gender disparity was observed, with male farmers being disproportionately affected compared with the other groups. Although no significant difference in age was noted, younger individuals appeared to be more susceptible. In addition, the disease was more prevalent among individuals with lower income and limited education (Table 1).

**Table 1:** Comparative analysis of socio-demographic, environmental, and lifestyle risk factors of heat stress nephropathy cases among 3 studied occupational groups viz., farmers, laborers, and others

Parameters	Farmers (*n* = 100)	Laborers (*n* = 32)	Other (*n* = 25)	*P*-values
Socio demography				
Age (yrs), mean ± SD	42.81 ± 13.01	49.81 ± 9.52	53.64 ± 12.13	1.46
No. of males	61	24	11	< 0.001^a^
No. of females	39	8	14	2.36
Level of education				
Illiterates	52	18	0	0.01
Primary	20	10	10	0.23
Degree	28	4	15	0.04
Income				
High	28	2	13	1.54
Average	34	8	10	2.30
Low	38	22	2	1.10
Environmental risk factors				
Without work > 4 mo/yr, %	20 (20)^b^	18 (56.25)	6 (24)	0.29
Heat exposure (h/d)	5.5 ± 2	3 ± 2	2 ± 1	< 0.001^a^
sWBGT, °C heat stress index	25.62 ± 0.12	21.23 ± 0.22	10.80 ± 2.53	< 0.001^a^
Agrochemical exposure, *n* (%)	96 (96)^b^	28 (87.5)	0 (0)	< 0.001^a^
Water intake (l/d)	2.68 ± 0.01	3.45 ± 0.21	3.48 ± 0.23	0.06
Sugary beverages intake (l/d)	2.2 ± 0.2	1.2 ± 0.01	0.5 ± 0.36	< 0.001^a^
Lifestyle-related risk factors				
h/o tobacco consumption *n* (%)	27 (27)	19 (59.3)	8 (32)	0.04
h/o alcohol consumption *n* (%)	53 (53)	22 (86.3)	6 (24)	0.02
NSAIDs > 3 mo *n* (%)	41 (41)	24 (75)	6 (24)	0.16

ANOVA, analysis of variance; h/o, history of; No., number; NSAID, nonsteroidal antiinflammatory drug; sWBGT, simplified wet bulb globe temperature.

Values are mean ± SD unless indicated otherwise.

a *P*-value for differences between groups: ANOVA for normally distributed continuous variables, Kruskal-Wallis for nonnormally distributed continuous variables, χ^2^ test for categorical variables.

b Significantly different from the other 2 categories in *post hoc* tests.

On average, farmers worked for 7.5 h/d in hot environments, whereas laborers had an effective work duration of approximately 5 h/d. Individuals in other occupational categories had the least exposure, working approximately 2 h/d (*P* < 0.001). Environmental heat exposure across the 3 groups was assessed using the sWBGT, which captured annual variations in workplace conditions. The highest mean sWBGT was recorded among agricultural workers, and it was significantly higher (*P* < 0.001**)** than for laborers, followed by individuals in other occupations. In addition, farmers had the highest exposure to agrochemicals compared with the other groups. Nephrotoxic pesticides such as chlorpyrifos, endosulfan, paraquat, and cypermethrin were commonly used in rice cultivation regions worldwide. There was no significant difference in daily water intake among the studied cohorts. However, farmers and laborers exhibited a higher intake of sugary beverages than individuals in other occupations, a finding that was statistically significant (*P* ≥ 0.05). Regarding lifestyle-related factors, alcohol, tobacco, and nonsteroidal antiinflammatory drug consumption were most prevalent among laborers, followed by farmers and individuals in other occupations. However, these differences were not statistically significant (Table 1).

### In Heat Stress Nephropathy Cases, Serological, and Hematological Parameters Varied Across Occupational Groups

The study assessed various clinical and biochemical parameters across 3 occupational groups, namely farmers, laborers, and individuals in other occupations. History of kidney stones was reported in 9.37% of laborers, compared with 2% of farmers and none in the other occupational group. Significantly low mean body mass index was observed among farmers compared with individuals of other occupation (*P* < 0.001). The proportion of individuals who fainted at work was highest among farmers (4.9%) compared with laborers (1.88%) and none in the other category. Dysuria was most common among farmers, followed by laborers and individuals in other occupations. Triglyceride levels were significantly higher among individuals in other occupations than in farmers and laborers (*P* < 0.001). Cholesterol, high-density lipoprotein, and low-density lipoprotein levels showed no statistically significant differences among the groups. Farmers had significantly (*P* < 0.001) higher hematocrit levels (48.70% ± 5.68%), followed by laborers (29.21% ± 6.37%) and the other group (21.97% ± 5.52%). Hemoglobin levels were significantly higher in laborers and farmers than in the other group. Erythrocyte counts were highest in farmers, whereas laborers and the other group had significantly lower values (*P* < 0.001). Platelet counts varied significantly across groups (*P* < 0.001), with the highest levels observed in the other occupational group (Table 2).

**Table 2:** Comparative analysis of medical history and clinical parameters of heat stress nephropathy cases among 3 studied occupational groups viz., farmers, laborers, and others

Parameters	Farmers (*n* = 100)	Laborers (*n* = 32)	Other (*n* = 25)	*P*-values
Perception results				
h/o Kidney stones, %	2	9.37	0	0.24
h/o UTI, %	16	12.5	16	1.68
Fainted at work, %	4.9	1.88	0	0.11
Dysuria, %	36	26.87	22.88	0.94
Systolic blood pressure, mean ± SD	118.34 ± 20.17	114.09 ± 13.04	115.04 ± 18.28	0.46
Diastolic blood pressure, mean ± SD	70.36 ± 15.21	67.78 ± 13.60	66.6 ± 17.17	0.455
Obesity (BMI ≥ 30 kg/m^2^) ,*n* (%)	20 (20)^a^	8 (25)	6 (24)	< 0.001^b^
Clinical results				
Blood glucose (mg/dl), mean ± SD	109.69 ± 27.74	106.18 ± 25.93	105.60 ± 20.41	0.15
Triglycerides (mg/dl), mean ± SD	138.89 ± 83.01^c^	124.91 ± 80.14	160.00 ± 74.51	< 0.001^b^
Cholesterol (mg/dl), mean ± SD	138.93 ± 37.93	132.09 ± 44.37	152.69 ± 37.29	0.14
HDL cholesterol (mg/dl), mean ± SD	40.85 ± 13.05	43.85 ± 15.22	44.34 ± 15.94	0.38
LDL cholesterol (mg/dl), mean ± SD	69.85 ± 25.68	69.64 ± 26.03	72.78 ± 23.23	0.86
Hematocrit %, Mean ± SD	48.70 ± 5.68^c^	29.21 ± 6.37	21.97 ± 5.52	< 0.001^b^
Hemoglobin (g/dl), mean ± SD	9.56 ± 1.85	9.71 ± 2.06	8.97 ± 1.87	< 0.001^b^
White cell counts/ul, mean ± SD	8.02 ± 2.09	7.98 ± 2.20	9.04 ± 3.69	0.15
% neutrophils, mean ± SD	5.09 ± 1.68	4.92 ± 1.43	5.87 ± 2.91	0.12
% Lymphocytes, mean ± SD	1.88 ± 0.60	2.088 ± 0.75	2.20 ± 0.81	0.06
Erythrocytes × 10^6^/ul, mean ± SD	7.74 ± 0.81^c^	3.95 ± 0.92†	3.54 ± 0.64	< 0.001^b^
Platelets × 10^3^/ul, mean ± SD	266.21 ± 80.95	258 ± 90.31	282.52 ± 99.62	< 0.001^b^

ANOVA, analysis of variance; BMI, body mass index; HDL, high-density lipoprotein; h/o, history of; LDL, low-density lipoprotein; UTI, urinary tract infection.

a Significant difference only between Farmers and others workers.

b *P*-value for differences between groups: ANOVA for normally distributed continuous variables, Kruskal-Wallis for not normally distributed continuous variables, χ^2^ test for categorical variables.

c Significantly different from the other 2 categories in *post hoc* tests.

### Prolonged Heat Stress Exposure Exacerbates Loss of Kidney Function and Dehydration Among Farmers

**Figure 3:**
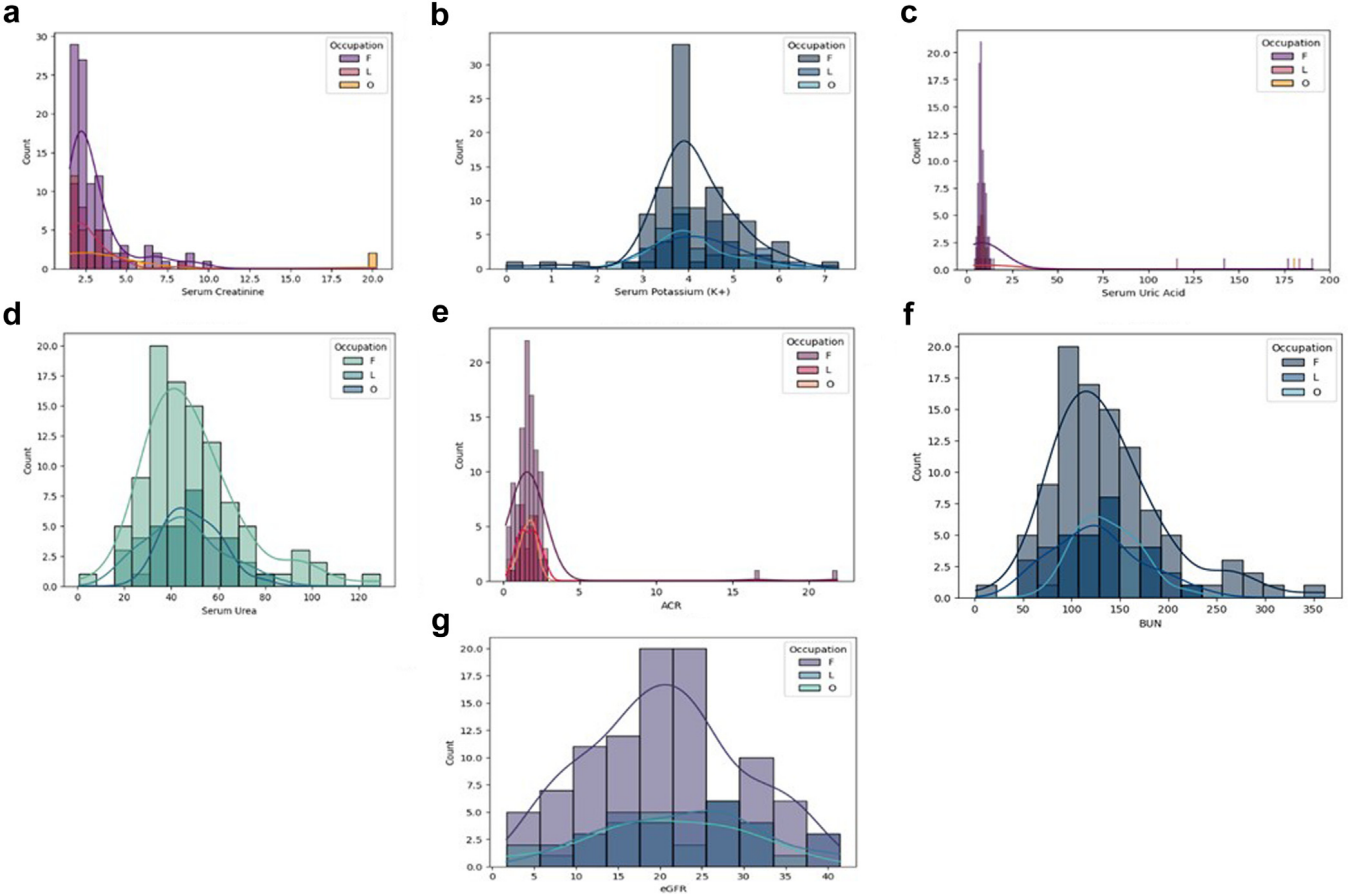
Plots representing comparison of renal functions of heat stress nephropathy cases (a) serum creatinine, (b) serum potassium, (c) serum uric acid, (d) serum urea, (e) albumin-to-creatinine ratio (ACR), (f) blood urea nitrogen (BUN), (g) estimated glomerular filtration rate (eGFR) among the occupational groups; F, farmer; L, labor; O, other.

The analysis of kidney function indicators revealed significant variations among the occupational groups. BUN levels were significantly higher in farmers than in laborers and individuals in other occupations (*P* < 0.001). The proportion of individuals with BUN levels > 20 mg/dl was also highest among farmers (15.8%), followed by laborers (8.2%) and other occupations (1.8%) (*P* = 0.015). Although SCr levels were elevated in all groups, no significant differences were observed across occupational cohorts. The prevalence of reduced eGFR (< 60 ml/min per 1.73 m^2^) was significantly higher in farmers (82%) than in laborers (71.87%) and other occupations (48%). Significant difference for prevalence of proteinuria > 30 mg/d was observed across the groups, with the highest in farmers (70%) followed by laborers (78.12%) and others (5%). Markers of dehydration were notably different across the groups. Farmers exhibited the highest U-SG and the highest proportion of individuals with urinary pH ≤ 5.5 compared with laborers and other occupations (*P* = 0.014). The BUN-to-SCr ratio > 20, indicative of dehydration, was significantly more prevalent in farmers than in laborers and other occupations (*P* < 0.001). HSPs, which are key biomarkers of cellular stress, were markedly elevated in farmers. Serum HSP27 levels were significantly (*P* < 0.001) higher in farmers than in laborers and other occupations. Urinary HSP27 and HSP70 concentrations were highest in farmers followed by laborers and individuals in other occupations. The fractional excretion of HSP70 was significantly high in farmers compared with laborers and other occupations (Table 3 and Figure 3).

**Table 3:** Biomarkers of kidney function and dehydration among workers in 3 occupations of heat stress nephropathy cases

Variables	Farmers (*n* = 100)	Laborers (*n* = 32)	Other (*n* = 25)	*P*-values
Indicators of kidney function				
BUN (mg/dl), mean ± SD	16.7 ± 5.1^a^	11.6 ± 2.51	7.77 ± 1.80	< 0.001^b^
BUN > 20 mg/dl %	15.8^a^	8.2	1.8	0.015^c^
SCr (mg/dl), mean ± SD	3.06 ± 1.79	2.78 ± 1.39	3.27 ± 3.67	0.388
SCr > 1.2 mg/dl, %	19.2	7.3	5.8	0.099
eGFR < 60 ml/min per 1.73 m^2^, *n*%	(82)82^a^	(23)71.87	(12) 48	< 0.001^b^
S-UA (mg/dl), Mean ± SD	48.71 ± 21.16	45.2 ± 16.17	20.72 ± 8.36	0.157
S-UA >7.2 mg/dl, %	16.72	5.50	1.48	0.544
Proteinuria > 30 mg/d, *n*%	70 (70)^a^	25 (78.12)	5 (20)	< 0.001^b^
eGFR < 60 ml/min per 1.73 m^2^, *n*% and Proteinuria > 30 mg/d, *n*%	65 (65)^a^	19 (59.37)	4 (16)	< 0.001^b^
Blood in urine, %	6.80	2.91	0	0.322
Indicators of dehydration				
Urinary specific gravity ≥ 1.0330%	26.32	22.86	9.45	0.129
Urinary pH ≤ 5.5%	30.22^a^	18.80	14.31	0.014^c^
BUN/SCr ratio > 20%	26.88^a^	4.24	0	< 0.001^b^
HSP27 mg/l (serum)	865^a^	678	615	< 0.001^b^
HSP27 mg/l (urine)	2087	1096	670	0.12
HSP70 mg/l (serum)	162	0	0	0.23
HSP70 mg/l (urine)	861^a^	365	291	< 0.001^b^
HSP70 fractional excretion	210.72/3^a^	35.68/8	1.73/5	< 0.001^b^

ANOVA, analysis of variance; BUN, blood urea nitrogen; eGFR, estimated glomerular filtration rate; HSP, heat shock protein; SCr, serum creatinine; S-UA, serum uric acid.

a Significant different from other 2 categories in *post hoc* test.

b Indicate significance difference at *P* ≤ 0.001.

c *P*-value for differences between groups: ANOVA for normally distributed continuous variables, Kruskal-Wallis for not normally distributed continuous variables, χ^2^ test for categorical variables.

### High Agricultural Intensity and Heat Stress Index Positively Correlated With Incidence of Renal Dysfunction in Studied Population

**Figure 4:**
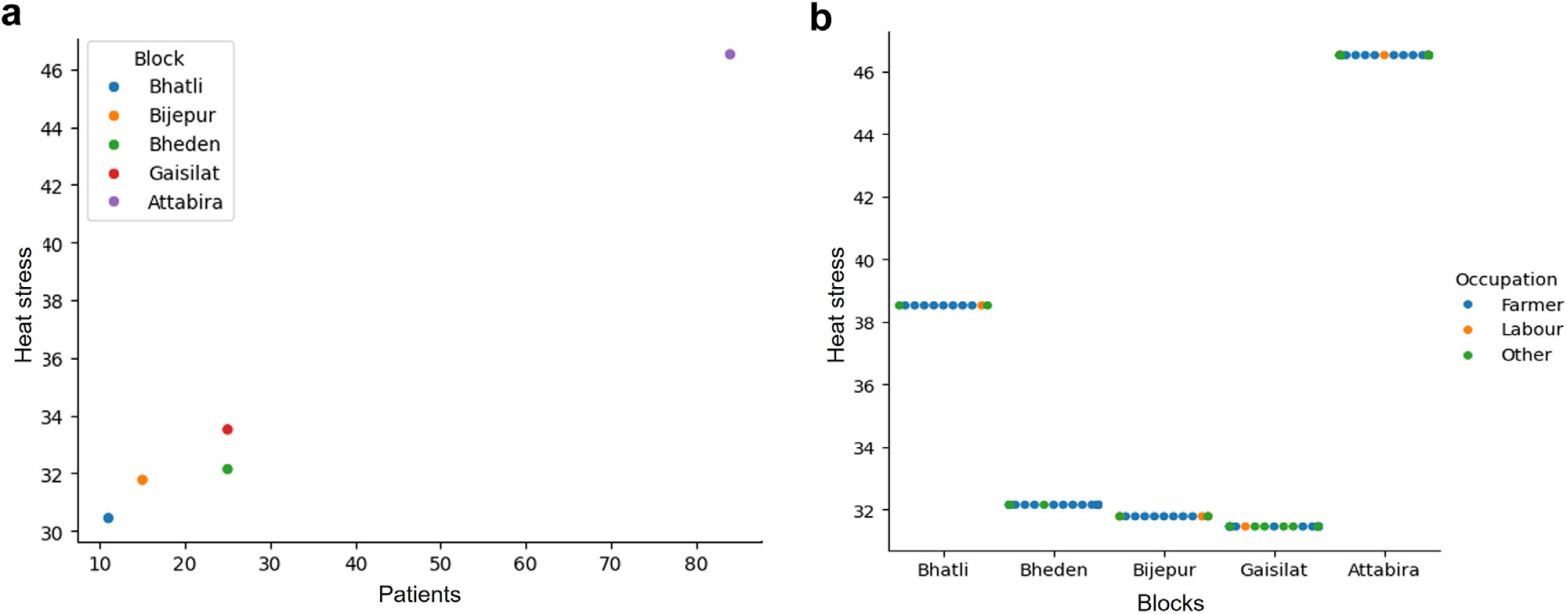
(a) Correlation between incidence of heat stress nephropathy and heat stress in hotspot areas of Bargarh district. (b) Correlation between incidence of heat stress nephropathy and heat stress in hotspot areas of Bargarh district grouped by occupation.

A positive correlation between the incidence of heat stress nephropathy and heat stress index was observed across hotspot villages. The highest number of cases (*n* = 95) was recorded in Attabira block, where the maximum heat stress exposure reached approximately 46 °C, surpassing all other studied regions. Gaisilat and Bheden blocks reported 26 cases each, with a heat stress range of 32 °C to 34 °C. Bijepur block exhibited the lowest incidence, with only 11 cases, where the heat stress level was recorded at 31.5 °C, the lowest among all hotspot blocks (Figure 4a). Across all study locations, individuals engaged in farming were the most exposed to heat stress and exhibited reduced kidney function. In Attabira block, where the estimated heat stress exposure was the highest (46.5 °C), 44 farmers were diagnosed with heat stress nephropathy, marking the highest prevalence among occupational groups. Laborers were the second most affected group, demonstrating a higher susceptibility to the disease compared to individuals in other occupations. The lowest prevalence of heat stress nephropathy was observed among farmers in the Bijepur block, where the heat stress index was minimal (31 °C) (Figure 4b).

### Biomarkers for Renal Disfunction Varied Between Heat Stress–Exposed and Nonexposed CKDu Cohorts

Out of 478 patients with CKDu, 157 were identified as being affected by heat stress nephropathy (heat stress–exposed group), whereas the remaining 321 patients were categorized as the heat stress–nonexposed group. A comparative account of biomarkers of renal dysfunction among both the heat stress–exposed and nonexposed groups are provided in Table 4. The comparative analysis reveals significant differences in kidney function and dehydration indicators between heat stress–exposed and nonexposed patients with CKDu. Exposed individuals showed significantly (*P* < 0.001) higher BUN, S-UA, and prevalence of eGFR < 60 ml/min per 1.73 m^2^. In addition, the presence of hematuria was significantly more frequent among exposed patients (*P* < 0.001). Advanced CKD stages (III and IV) were more common in both heat-exposed and nonexposed groups. Although SCr was higher in the exposed group, the difference was not statistically significant (*P* = 0.371). Dehydration markers such as U-SG ≥ 1.033, low urinary pH, elevated BUN-to-SCr ratio (> 20), and HSP27 and HSP70 in both serum and urine was significantly high in the heat-exposed group. These findings suggest that heat stress exacerbates both renal dysfunction and dehydration, potentially accelerating CKDu progression.

**Table 4:** Comparative account of indicators of kidney function and dehydration between heat stress–exposed and heat stress–nonexposed CKDu patients

Variables	Heat stress–exposed CKDu patients (*n* = 157)	Heat stress–nonexposed CKDu patients (*n* = 321)	*P*-values
Indicators of kidney function			
BUN (mg/dl), mean ± SD	21.02 ± 3.13^a^	10.23 ± 7.08	< 0.001^b^
SCr (mg/dl), mean ± SD	6.03 ± 2.28	4.04 ± 0.34	0.371
eGFR < 60 ml/min per 1.73 m^2^, *n* (%)	134 (85.35)	96 (29.9)	< 0.001^b^
S-UA (mg/dl), mean ± SD	38.21 ± 15.23^a^	28.63 ± 15.16	< 0.001^b^
Proteinuria > 30 mg/d	46.87	43	0.069
Blood in urine, %	26.38^a^	7.54	< 0.001^b^
CKD stage I, *n* (%)	0 (0)	0 (0)	-
CKD stage II, *n* (%)	23 (14.64)	86 (26.79)	-
CKD stage III, *n* (%)	59 (37.57)	105 (32.71)	-
CKD stage IV, *n* (%)	49 (31.21)	98 (30.52)	-
CKD stage V, *n* (%)	26 (16.56)	32 (9.96)	-
Indicators of dehydration			
Urinary specific gravity ≥ 1.0330%	25.12^a^	10.75	< 0.001^b^
Urinary pH ≤ 5.5%	27.15	12.40	0.012
BUN/SCr ratio > 20%	31.01^a^	7.03	< 0.001^b^
HSP27 mg/l (serum)	721^a^	108	< 0.001^b^
HSP27 mg/l (urine)	1602^a^	1029	< 0.001^b^
HSP70 mg/l (serum)	97^a^	0	< 0.001^b^
HSP70 mg/l (urine)	739^a^	204	< 0.001^b^

ANOVA, analysis of variance; BUN, blood urea nitrogen; CKD, chronic kidney disease; eGFR, estimated glomerular filtration rate; HSP, heat shock protein; SCr, serum creatinine; S-UA, serum uric acid.

a Significant different from other 2 categories in *post hoc* test.

b Indicate significance difference at *P* ≤ 0.001.

### Renal Histopathology Reveals Chronic Tubulointerstitial Nephritis

**Figure 5:**
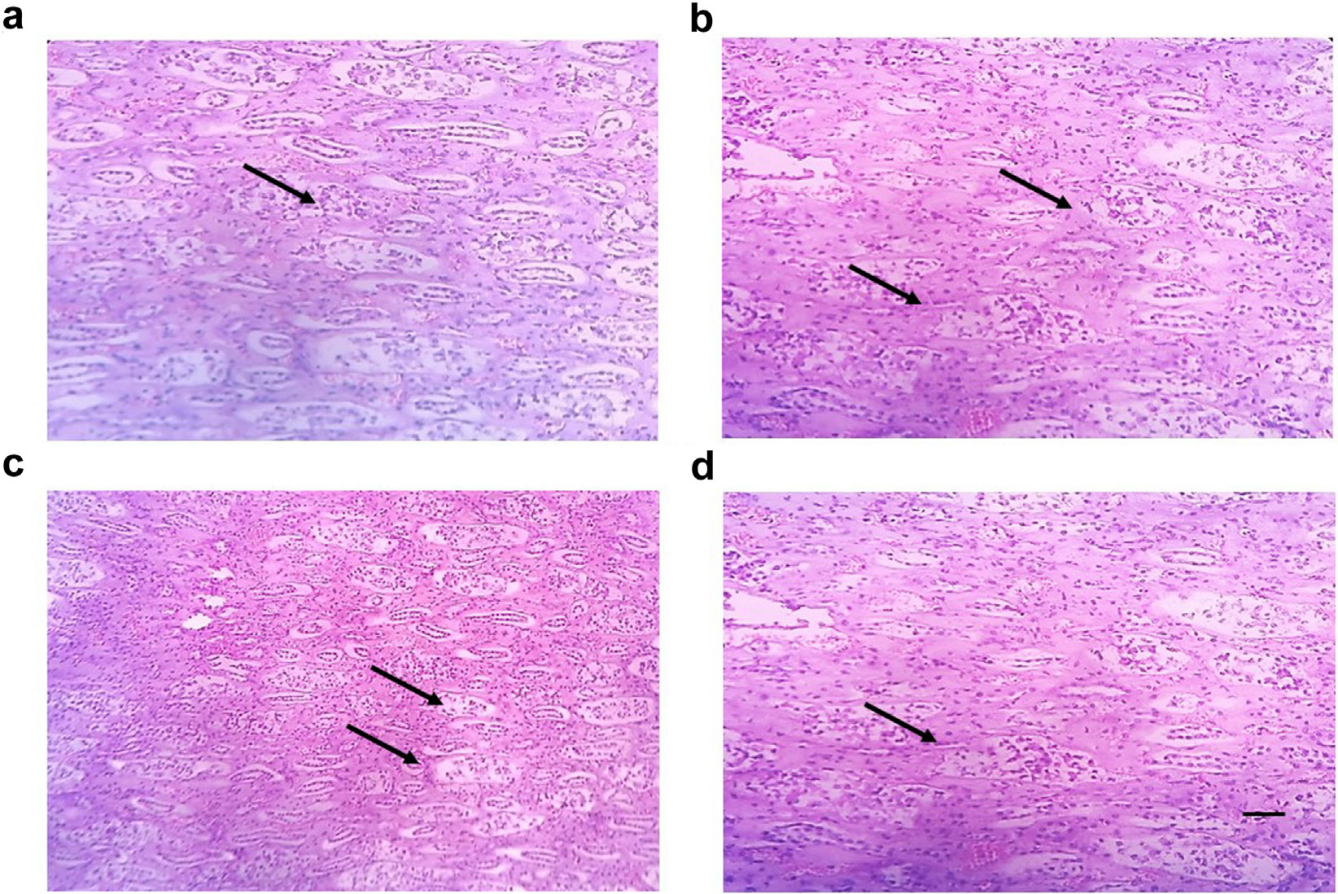
Representative figures showing the presence of fibroblast cells in renal histopathology slides of 4 representative patients suffering from tubulointerstitial nephritis.

Microscopic analysis of renal biopsy samples from 8 subjects revealed substantial infiltration of inflammatory cells, including lymphocytes, histiocytes, plasma cells, and scattered neutrophils within the interstitium and tubules. These findings are characteristic of chronic tubulointerstitial nephritis. Furthermore, focal regions exhibited the presence of fibroblasts, indicating early-stage tubular fibrosis. These histopathological alterations highlight the persistent inflammatory response and structural remodeling associated with progressive renal damage in the affected individuals (Figure 5).

## Discussion

Heat stress and cyclic dehydration have been potentially linked to CKDu in many parts of tropical countries.^23^ A recent study carried out by our laboratory has identified 16 hotspot villages reporting higher prevalence of CKDu in Bargarh district, Odisha. All these hotspot villages fall under an intense agricultural zone of the district. In the current study, we found that areas having higher heat stress index had greater prevalence of heat stress nephropathies. Similar studies from central America, Sri Lanka, and Uddanam province of South India had also reported higher prevalence of heat stress nephropathy cases in areas with dry climatic zones.^20^ The increased prevalence of CKDu in warm tropical countries has added credence to the hypothesis of recurrent heat stress and dehydration as a leading cause of kidney disease.

Farming in Bargarh district is largely informal, with minimal occupational health regulations. Many farmers work long hours in extreme temperatures without adequate hydration, increasing the risk of dehydration-induced kidney injury. Socioeconomic constraints and poor access to clean drinking water in rural farming communities further intensify the risk. Our survey indicates that few farmers use protective clothing, such as hats, long sleeves, or cooling garments, because of discomfort, cost, and lack of awareness. Exposure to agrochemicals in combination with heat stress may accelerate kidney damage. The chronic nature of exposure, with years of cumulative heat stress and prolonged work hours under direct sun, likely contributes to the high prevalence of heat stress nephropathy among farmers. Compared with other occupational groups, farmers have longer, uninterrupted exposure to high environmental temperatures, often exceeding 8 to 10 h/d during peak seasons.^6^

Multiple studies have implicated environmental toxicants such as agrochemicals, heavy metals, and fluoride in the development of CKDu, as observed in Sri Lankan Agricultural Nephropathy.^21^ In contrast, heat stress and dehydration are proposed as primary drivers of Mesoamerican nephropathy and other chronic interstitial nephritis in agricultural communities epidemics.^24^^,^^25^ Prolonged dehydration from strenuous labor in high temperatures can trigger AKI, hyperosmolarity, and elevated vasopressin levels, all of which contribute to CKD progression. It can also cause hyperuricemia and rhabdomyolysis, exacerbating tubulointerstitial damage.^26^ Although some evidence supports the heat stress/dehydration hypothesis and links it to climate change, this remains debated. Critics argue that current data are insufficient to establish cyclical dehydration as the sole cause of chronic interstitial nephritis in agricultural communities, with studies showing that temperature alone may not significantly influence Mesoamerican nephropathy incidence when adjusted for agricultural practices. Moreover, the modest global temperature rise—approximately 0.4 °C from 1960 to 1990—seems insufficient to fully account for the rapid and severe onset of the epidemic in affected communities.

In this study, farmers showed a greater degree of exposure to heat stress, which may be due to prolonged work period in field and had marked dehydration and kidney dysfunction. To a lesser degree, reduced kidney function was noted among laborers but comparatively much less among individuals with other occupation. The present study site shares a similar agroclimatic environment with Uddanam of Andhra Pradesh, India. Previous works reported increased cases of heat stress nephropathy among agricultural workers in Uddanam, typically termed as Uddanam nephropathy.^27^ Individuals practicing farming showed significantly high sWBGT index, suggesting greater exposure to heat stress mediated by occupational heat exposure within the working environment. Farmers work at a faster pace, have lesser exposure to shade and report more weight loss and fainting episodes. Shorts breaks are taken during work hours, but still under sun. The mean temperature and WBGT are already very high in the morning in tropical countries. As per the recommendation of US OSHA, there is a need for rest about 50% of the time when WBGT exceeds 28°C, to avoid increased core body temperature.^28^ Farmers usually do not take such breaks, which could be a possible cause of the higher WBGT index among them. To counteract heat production and maintain body temperature, the body usually undergoes profuse sweating, which could lead to dehydration.

In the current study, no significant difference was found in the amount of fluid intake among the 3 groups, but marked difference was noted for intake of sugary beverages. Farmers self-reported, high fructose fluid intake during work hours. Sugary beverages that contain fructose are known to increase the risk of albuminuria and have been reported to induce renal injury in laboratory animals.^22^ A previous study by our laboratory in these hotspot areas found a possible linkage of agrochemical and heavy metal exposure as driving causes for CKDu.^23^ Several other studies in different parts of the globe have reported environmental risk factors such as pesticide and heavy metal exposure, excessive fructose intake, consumption of locally made alcohol as a potent risk factor for CKDu. Farmers are directly exposed to agrochemicals in the field and are possibly more susceptible to the development of renal disfunction or CKDu. Studies in Sri Lanka and Uddanam have identified heat exposure and associated dehydration tendency in agricultural workers as an underlying cause for CKDu in those affected areas. Daily exposure to heat stress and dehydration may cause repeated renal hypoperfusion episodes, and intermittent subclinical rhabdomyolysis associated with excessive excretion leading to repeated AKI through the release of inflammatory mediators, including oxidants, cytokines, and uric acid, that gradually lead to CKD. In the current study, renal function was significantly reduced; about 2-fold in agricultural workers compared with the other 2 groups, which was evident from marked decrease in eGFR. The increase in SCr level, BUN, and uric acid is probably an effect of reduced eGFR during strenuous work in the agricultural fields. In addition, increased muscle creatinine and purine breakdown leads to increased azotemia.^29^ Work in hot environment redistributes blood flow to muscles and skin to increase heat loss. This physiological phenomenon decreases blood flow to visceral organs such as the intestines and kidneys. The elevated hematocrit levels observed in farmers and laborers could be attributed to dehydration induced by their hot working conditions. Urine pH was significantly lower in farmers than in the other studied cohorts. This might reflect the effect of hypovolemia and lactic acid generation due to dehydration. Significantly increased U-SG among farming communities may be due to excessive water loss and decreased central blood flow, which activates renin angiotensin aldosterone system, resulting in release of aldosterone. Aldosterone increases renal tubular reabsorption of water and sodium, leading to increased urine osmolality and U-SG. Several other studies have reported similar increased U-SG in field workers exposed to heat.^8^

Interestingly, S-UA level elevated significantly among farmers followed by laborers in the current study; which find support from a study in Mesoamerica that reported increased S-UA levels among sugarcane cutters.^29^ Heat stress is known to raise S-UA levels, in part from subclinical rhabdomyolysis^17^ but also from renal hypoperfusion. Moreover, hyperuricemia is a well-known factor for CKD. The formation of acidic urine due to lactic acid production is proposed as a contributing factor increasing the risk of urate crystal formation, potentially associated with strenuous exercise and prolonged exposure to heat stress. In line with the present finding, increased albumin-to-creatinine ratio level was noted among agricultural workers in 4 villages of the North Central Province. Participants of 3 villages from the North Central Province of Sri Lanka with high burden of CKDu showed higher incidence of dehydration symptoms and albuminuria (albumin-to-creatinine ratio > 30 mg/g) than participants from 1 village with lower cases of CKDu.^7^

We observed significantly higher levels of HSP27 and HSP70 in the serum of farmers and laborers. The presence of these HSPs in the bloodstream results from increased production and release by damaged cells, compounded by reduced renal clearance in individuals with kidney failure.^14^ The heat shock response is activated by an upregulation of HSPs during periods of heightened stress, such as elevated urea levels or hypoxia, aiming to preserve cell integrity and minimize cell death.^30^ In CKD, factors such as increased cell death, necrosis due to uremic toxins, inflammation, and oxidative stress can prompt the release of intracellular HSPs, leading to elevated serum concentrations.

Renal histopathological findings in 8 selected patients from the present study elucidated the pathogenesis of tubulointerstitial nephritis and focal tubular fibrosis in few cases. Dehydration due to heat stress is believed to be a primary cause for tubulointerstitial nephritis. Experimental evidence in rats have revealed that repeated exposure to heat stress causes decline in renal function that gradually damages tubulointerstitial cells resulting in tubulointerstitial nephritis.^4^ When the body is exposed to high temperature, especially in hot and humid environment or during strenuous physical activity, dehydration can occur rapidly. Dehydration leads to renal hypoperfusion, which compromises kidney function. The reduced blood flow to the kidneys results in inadequate oxygen and nutrient delivery to the renal tubular cells, leading to cellular injury and dysfunction.^12^ It triggers an inflammatory cascade, releasing cytokines and chemokines that directly harm renal tubular cells and the interstitium. Exposure to heat stress, causes rhabdomyolysis that releases myoglobin into the bloodstream, which is then filtered by the kidneys. Myoglobin can accumulate in renal tubules, causing obstruction and injury, known as myoglobinuric AKI; a significant factor for tubulointerstitial nephritis in heat stress nephropathy.^13^^,^^15^ Chronic or severe cases of heat stress nephropathy can lead to persistent renal inflammation and fibrosis, ultimately leading to progressive kidney dysfunction.

A previous study from our laboratory identified 7 different nephrotoxic pesticide residues such as dichloro-diphenyl-trichloroethane, chlorpyrifos, endosulfan, malathion, carbofuran, paraquet, and cypermethrin above the permissible limit in different environmental compartments such as soil, water, and rice grain.^22^ The direct and indirect exposure to these pesticides could possibly cause renal dysfunction. Farming communities are occupationally exposed to agrochemicals and heat stress simultaneously as part of their activities in crop fields. However, interactions between these pesticides and environmental conditions such as heat stress and their association with the onset of CKD in the Indian population is poorly investigated. In the present study, higher prevalence of CKDu among farming communities in the blocks with high intensity of agricultural activities in combination with elevated heat stress indicates possible association of pesticide exposure and cyclic dehydration in disease onset and progression. Supporting our findings, studies carried out on Sri Lankan agricultural nephropathy reported higher prevalence of CKDu among farming communities, which could be linked to cyclic dehydration, direct exposure to pesticide residues, and heat.^16^, ^17^, ^18^ Further mechanistic study exploring involvement of possible pathways and signaling molecules responsible for heat stress nephropathy is necessary to understand the disease progression and management in the community.

However, there are several limitations of the present study. First, it is cross-sectional in nature, which limits its ability to establish causal relationships between heat stress and CKDu. Future research should focus on long-term cohort studies to track disease progression and identify early biomarkers of heat stress nephropathy. Further, the interplay between heat stress, dehydration, agrochemical exposure, and other environmental or genetic factors remains unclear in the present study. Although HSPs and oxidative stress markers have been implicated in CKDu, the exact pathophysiological mechanisms remain poorly understood. Therefore, though the findings from Bargarh district is significant, it may not fully represent CKDu patterns in other agroclimatic regions.

## Conclusion

Considering the present study, it can be concluded that the cases of heat stress nephropathy were hiked in areas reporting higher heat stress index, establishing a positive correlation between CKDu incidences and heat stress index in the study area. Individuals practicing farming were occupationally more exposed to heat conditions than the other 2 occupational groups and therefore reported higher incidences of heat stress nephropathy cases. HSP concentrations were found to be 2- to 3-fold higher among farmers, which suggests the underlying case of chronic heat exposure. Renal histopathological examinations of selected patients revealed tubulointerstitial nephritis, consistent with heat stress–induced nephropathy. These findings highlight the urgent need for comprehensive occupational health interventions, including improved hydration strategies and work-rest schedules aligned with heat stress guidelines. Addressing these challenges is crucial not only for the health and well-being of agricultural workers but also for advancing our understanding of environmental and health research. However, the synergistic effects of environmental toxins such as pesticides and heavy metals along with heat stress could be a possible reason for increased CKDu incidences among agricultural practitioners that needs further comprehensive study.

## Disclosure

All the authors declared no competing interests.

## Patient Consent

Informed consent was obtained from the individuals who participated in the study.

## Supplementary Material

Supplementary File (PDF) **Figure S1.** Representative ultrasonography of participants with kidney stone. **Table S1.** Questionnaire used in the study. **Table S2.** Details of socio-demography, Environmental and Lifestyle related risk factors of CKDu patients in studied population. **Table S3.** Hematological and serological details of the CKDu patients in the studied population. **Table S4.** Details of the Indicators of Kidney Dysfunction and Dehydration.
