# Supplementary material for: Heat Stress Nephropathy in CKD of Uncertain Etiology Hotspots of Bargarh District Odisha, India
Source: Kidney Int Rep. 2025 Jul 15;10(10):3379–94. doi: 10.1016/j.ekir.2025.07.013 (PMC12546656; doi:10.1016/j.ekir.2025.07.013)
Supplement: Supplementary File (PDF) — Figure S1. Representative ultrasonography of participants with kidney stone. Table S1. Questionnaire used in the study. Table S2. Details of socio-demography, Environmental and Lifestyle related risk factors of CKDu patients in studied population. Table S3. Hematological and serological details of the CKDu patients in the studied population. Table S4. Details of the Indicators of Kidney Dysfunction and Dehydration. [file mmc1.pdf]

Supplementary Table S1. Questionnaire used in the study.

ପ୍ରକଳ୍ପର ଶୀର୍ଷକ/ Title of the Project

ଓଡ଼ିଶାର ବରଗଡ଼ ଜିଲ୍ଲାରେ ଗରମ ଚାପ ନେଫ୍ରୋପାଥି ସମ୍ବନ୍ଧରେ ଦୀର୍ଘସ୍ଥାୟୀ ବୃକକ୍ ରୋଗର ଅଧ୍ୟୟନ ।
Study of Chronic Kidney Diseases with respect to heat stress nephropathy in Bargarh District, Odisha.

ଅଧ୍ୟୟନ ବିଷୟ ପାଇଁ ପ୍ରଶ୍ନାବଳୀ
Questionnaire for study subjects

ପୁରା ନାମ<br>Full Name	
ଲିଙ୍ଗ<br>Gender	
ବୟସ<br>Age	
ଉଚ୍ଚତା<br>Height	
ଓଜନ<br>Weight	
ବୃତ୍ତି<br>Occupation	
ଠିକଣା<br>Address	
ଫୋନ୍ ନମ୍ବର<br>Tel. No	
ବୃକକ୍ ରୋଗ ନିରୁପଣ ଆବିଷ୍କାର ହୋଇଥିବା ବୟସ<br>Age at which kidney disease was discovered	
ଆପଣଙ୍କୁ କେବେ ତାପ ଜନିତ ରୋଗ ହୋଇଥିବାର ଚିହ୍ନଟ ହୋଇଛି କି?<br>Have you ever been diagnosed with heat-related illnesses before?	
ଆପଣଙ୍କର ଅନ୍ୟ କୌଣସି ଦୀର୍ଘକାଳୀନ ରୋଗ ଅଛି କି?<br>Do you have any other chronic medical conditions?	
ଆପଣ କ'ଣ ନିୟମିତ ଭାବରେ କୌଣସି ଔଷଧ ଖାଉଛନ୍ତି କି?<br>Are you taking any medications regularly?	
ମଧୁମେହ ରୋଗୀ କି ନାହିଁ?<br>Diabetic or not	
*ପ୍ରାରମ୍ଭିକ ଲକ୍ଷଣ ଯଦି ଥାଏ<br>*Starting symptoms if any	
ରୋଗ ନିର୍ଣ୍ଣୟ କିପରି କରାଯାଇଥିଲା ?<br>How was diagnosis made ?	
ଆପଣ କ'ଣ ଗରମ ପରିବେଶରେ କାମ କରନ୍ତି କିମ୍ବା ଅଧିକ ସମୟ ବିତାଇଥାନ୍ତି?<br>Do you work or spend extended periods in hot environments?	

ଆପଣ ସାଧାରଣଃ କେତେଥର ଉଚ୍ଚ ତାପମାତ୍ରାର ସମ୍ମୁଖୀନ ହୁଅନ୍ତି?<br>How often are you exposed to high temperatures?	
ଗରମ ପାଗରେ କିମ୍ବା ଗରମ ପରିବେଶରେ କାମ କରିବା ସମୟରେ ଆପଣ ଥଣ୍ଡା ରହିବା ପାଇଁ ପଦକ୍ଷେପ ନିଅନ୍ତି କି?<br>Do you take measures to stay cool during hot weather or while working in hot environments?	
ଆପଣ କ'ଣ ସୁରକ୍ଷା ପୋଷାକ ପିନ୍ଧନ୍ତି ? (ହଁ କିମ୍ବା ନା)<br>Do you Wearing protective clothing? (Yes or No)	
ଆପଣ ସାଧାରଣତଃ ଗୋଟିଏ ଦିନରେ କେତେ ପରିମାଣରେ ପାଣି ପିଅନ୍ତି?<br>How much water do you typically drink in a day?	
ତରଳ ପଦାର୍ଥ ଏବଂ ଫ୍ରୁକ୍ଟୋଜ୍ ସେବନ (କାର୍ଯ୍ୟ ଦିବସ)<br>Fluid and Fructose intake(workday)	
ତରଳ ପଦାର୍ଥ ଏବଂ ଫ୍ରୁକ୍ଟୋଜ୍ ସେବନ (କାର୍ଯ୍ୟ ଦିବସ ବ୍ୟତୀତ)<br>Fluid and Fructose intake(non-workday)	
ଆପଣ ବର୍ତ୍ତମାନ ଆପଣଙ୍କର ତରଳ ପଦାର୍ଥ ଗ୍ରହଣରେ କୌଣସି ପରିବର୍ତ୍ତନ ଲକ୍ଷ୍ୟ କରିଛନ୍ତି କି?<br>Have you noticed any changes in your fluid intake recently?	
କାର୍ଯ୍ୟ ସମୟରେ ଆଘାତ କିମ୍ବା ଦୁର୍ଘଟଣା<br>Injuries or accidents during work experience	
ଆପଣ କ'ଣ ନିୟମିତ ଭାବରେ କ୍ୟାଫିନ୍ କିମ୍ବା ଆଲକୋହଲ ଯୁକ୍ତ ପାନୀୟ ସେବନ କରନ୍ତି?<br>Do you consume beverages containing caffeine or alcohol regularly?	
ଆପଣଙ୍କ କର୍ମକ୍ଷେତ୍ରରେ ଗରମ ପ୍ରଭାବକୁ କମାଇବା ପାଇଁ କୌଣସି ପଦକ୍ଷେପ ଅଛି କି?<br>Are there any measures in place to mitigate heat exposure at your workplace?	
ଗରମ ସମୟରେ ଆପଣ କେତେଥର ବିଶ୍ରାମ ଏବଂ ଜଳୀୟ ଅଂଶ ଗ୍ରହଣ କରିବା ପାଇଁ ବିରତି ନିଅନ୍ତି?<br>How often do you take breaks to rest and hydrate during exposure to heat?	
ଗରମ ଅବସ୍ଥାରେ କାମ କରିବା ପରେ ଆପଣ କ'ଣ ଆପଣଙ୍କ ପରିସ୍ରାର ରଙ୍ଗ କିମ୍ବା ପରିମାଣରେ ପରିବର୍ତ୍ତନ ଲକ୍ଷ୍ୟ କରନ୍ତି (ଗାଢ଼ ପରିସ୍ରା କିମ୍ବା ପରିସ୍ରାର ମାତ୍ରା କମ ହେବା)?<br>Do you notice a change in your urine colour or volume after working in hot conditions (Darker urine or reduced urine output)?	
ଗରମ ସଂସ୍ପର୍ଶରେ ଆସିବା ପରେ ଆପଣ କ'ଣ ବାରମ୍ବାର କିମ୍ବା ଅବ୍ୟକ୍ତ ପିଠି କିମ୍ବା ବୃକକ୍ ଯନ୍ତ୍ରଣା ଅନୁଭବ କରନ୍ତି?<br>Do you experience frequent or unexplained back or kidney pain after exposure to heat?	

ଆପଣ କେବେ ଗରମ ପରିବେଶରେ ଅସୁସ୍ଥ ଅନୁଭବ କରି ଡାକ୍ତରୀ ପରାମର୍ଶ ପାଇଁ ଯାଇଛନ୍ତି କି?<br>Have you ever shout medical attention often feeling unwell in a hot environment?	
ଯେକୌଣସି ବୃକକ୍ ଷ୍ଟୋନ୍<br>Any kidney Stones	
ଯେକୌଣସି ବାରମ୍ବାର ମୂତ୍ର ସଂକ୍ରମଣ<br>Any frequent urinary infections	
ଆପଣ କ'ଣ ଗରମ ପରିବେଶରେ କୌଣସି କଠିନ ଶାରୀରିକ କାର୍ଯ୍ୟକଳାପ କିମ୍ବା ପରିଶ୍ରମ କରିଛନ୍ତି?<br>Have you engaged in any strenuous physical activity or exertion in hot conditions recently?	
ଆପଣଙ୍କ ଅଞ୍ଚଳରେ ସମ୍ପ୍ରତି କୌଣସି ଅତ୍ୟଧିକ ଗରମ ପରିବେଶର ସମ୍ମୁଖୀନ ହୋଇଛନ୍ତି କି ?<br>Have there been any extreme heat events in your area recently?	
ଯେକୌଣସି ରକ୍ତ ଅନ୍ତଃଭରଣ କେବେ କରିଛନ୍ତି କି ?<br>Any blood transfusions	
ଯେକୌଣସି ଲାବ୍ ଫଳାଫଳ ଉପଲବ୍ଧ ଅଛି କି ?<br>Any labs result available	
ଯେକୌଣସି ଏକ୍ସ-ରେ କିମ୍ବା ଅଲ୍ଟ୍ରାସାଉଣ୍ଡ ରିପୋର୍ଟ<br>Any X-rays or Ultrasound reports	
ଯେକୌଣସି ବୃକକ୍ ବାୟୋପ୍ସି ରିପୋର୍ଟ ଅଛି କି ?<br>Any kidney Biopsy report	
ଯଦି ମହିଳା, ଗର୍ଭାବସ୍ଥାରେ କୌଣସି ସମସ୍ୟା ହୋଇଥିଲା କି ?<br>If female, any problems during pregnancy(ies)	
ପରିସ୍ରାରେ ପୃଷ୍ଟିସାର କିମ୍ବା ରକ୍ତର କୌଣସି ଇତିହାସ ଅଛି କି ?<br>Any history of protein or blood in urine	
ନିୟମିତ ସେବନ କରୁଥିବା ଯେକୌଣସି ଔଷଧର ନାମ (ଗୁଡିକ)<br>Name(s) of any medication(s) the person is taking	

* ଲକ୍ଷଣ/ Symptoms:

ଆପଣ ସମ୍ପ୍ରତି ନିମ୍ନଲିଖିତ ଲକ୍ଷଣଗୁଡ଼ିକ ମଧ୍ୟରୁ କୌଣସି ଅନୁଭବ କରିଛନ୍ତି କି? (ପ୍ରଯୁଜ୍ୟ ସମସ୍ତ ଯାଞ୍ଚ କରନ୍ତୁ)

Have you experienced any of the following symptoms recently? (Check all that apply)

- ଥକାପଣ (Fatigue)

- ମାଂସପେଶୀ କ୍ରାମ୍ପ (Muscle cramps)

- ବାନ୍ତି କିମ୍ବା ବାନ୍ତି ହେବା (Nausea or vomiting)

- ମୁଣ୍ଡବିନ୍ଧା (Headache)

- ମୁଣ୍ଡ ବୁଲାଇବା (Dizziness)

- ପରିସ୍ରା କମ ହେବା (Decreased urine output)

- ଗାଢ଼ ରଙ୍ଗର ପରିସ୍ରା (Dark-colored urine)

- ଦ୍ୱନ୍ଦ୍ୱ କିମ୍ବା ଦିଗଭ୍ରାନ୍ତ ହେବ (Confusion or disorientation)

- ଦୁର୍ବଳତା (Weakness)

ଟିପ-ଚିହ୍ନ / ଦସ୍ତଖତ
Thumb Impression / Signature

ମୁଖ୍ୟ ଅନୁସନ୍ଧାନକାରୀଙ୍କ ଦସ୍ତଖତ
Signature of the Principal Investigator

Supplementary Table S2. Details of socio-demography, Environmental and Lifestyle related risk factors of CKDu patients in studied population.

Parameters	Farmers (N=361)	Laborers (N=72)	Other (N=45)	P Values
(A) Socio demography				
Age (years) Mean±SD	47.91 ± 10.02	58.21 ± 9.31	51.78 ± 11.10	1.36
No. of Males	281	43	28	<0.001*
No. of Females	80	29	17	2.32
Level of Education				
Illiterates	216	31	0	0.01
Primary	101	33	16	0.21
Degree	44	8	29	0.03
Income				
High	98	6	27	1.55
Average	111	11	13	2.32
Low	152	55	5	1.12
**(B) *Environmental Risk Factors***				
Without work > 4 months/years %	72 (19.94)	12 (16.66)	3 (16.6)	0.27
Heat Exposure (hours/day)	6± 1	4± 0.5	2 ±1	<0.001*
sWBGT/ ^{0}C Heat Stress Index	118.96 ± 0.31 †	64.71 ± 0.28 †	22.90 ± 2.66	<0.001*
Agrochemical Exposure n(%)	357 (98.8) †	16 (22.2) †	0 (0)	<0.001*
Water intake (L/day)	3.92 ± 0.05	3.81 ± 0.32	4.61 ± 0.22	0.07
Sugary Beverages intake (L/day)	1.1± 0.1	1.6± 0.01	2.0 ± 0.36	0.06
**(C) *Life style related risk factors***				
h/o Tobacco Consumption n(%)	216 (59.8)	34 (47.2)	11 (24.4)	0.09
h/o alcohol Consumption n(%)	97 (26.8)	52 (72.2)	8 (17.7)	0.04
NSAIDs > 3 months n(%)	146 (40.4)	61 (8.3)	4 (8.8)	0.17

Values are mean±SD unless indicated otherwise.

*p Value for differences between groups: ANOVA for normally distributed continuous variables, Kruskal-Wallis for not normally distributed continuous variables, $\chi 2$ test for categorical variables.

†Significantly different from the other two categories in post hoc tests.

Supplementary Table S3. Haematological and serological details of the CKDu patients in the studied population.

**Parameters**	**Farmers (N=361)**	**Laborers (N=72)**	**Other (N=45)**	**P Values**
(A). Perception Resultss				
h/o Kidney stones, n (%)	7 (1.93)	20 (27.7)	0 (0)	0.27
h/o UTI, n%	57.76	9	7.2	0.06
Systolic Blood Pressure Mean±SD	109.27 ±23.81	111.05 ± 12.01	112.05 ± 17.92	0.37
Diastolic Blood Pressure Mean±SD	61.23 ± 19.22	65.84 ± 15.01	68 ± 21.04	0.43
Obesity (BMI ≥30 kg/m^2) N (%)	78 (21.06)§	26 (36.11)	28(62.22)	<0.001*
Dysuria %	129.96	60.66	39.22	0.93
(B). Clinical Results				
Blood glucose (mg/dl) Mean±SD	122.35 ± 54.02	145.80 ± 69.93	121 ± 25.35	0.17
Triglycerides (mg/dl) Mean±SD	141.05 ± 82.40†	138.37 ± 78.20	133.06 ± 82.45	<0.001*
Cholesterol (mg/dl) Mean±SD	142.39 ± 40.53	146.08 ± 41.84	152.69 ± 37.29	0.11
Hematocrit % Mean±SD	28.43 ± 5.87†	24.76 ± 5.37	20.63 ± 5.60	<0.001*
Hemoglobin (g/dl) Mean±SD	9.52 ± 1.89	9.42 ± 1.72	6.02 ± 1.33	<0.001*
White Cell Counts/uL Mean±SD	8.14 ± 2.43	8.02 ± 2.44	8.36 ± 2.43	0.18
% neutrophils Mean±SD	5.14 ± 1.88	5.03 ± 1.89	5.24 ± 2.08	0.21
% Lymphocytes Mean±SD	1.98 ± 0.66	2.01 ± 0.65	1.9 ± 0.57	0.07

Values are mean±SD unless indicated otherwise.

*p Value for differences between groups: ANOVA for normally distributed continuous variables, Kruskal-Wallis for not normally distributed continuous variables, $\chi 2$ test for categorical variables.

†Significantly different from the other two categories in post hoc tests.

§Significant difference only between Farmers and others workers.

HDL, high density lipoprotein; LDL, low density lipoprotein

Supplementary Table S4. Details of the Indicators of Kidney Dysfunction and Dehydration

Variables	Farmers (N=361)	Laborers (N=72)	Other (N=45)	P Values
**(A) Indicators of Kidney function**				
BUN (mg/dL), Mean ± SD	22.36 ± 8.36	22.72 ± 7.08	23.87 ± 9.59	<0.001**
Serum Creatinine (SCr) (mg/dL), Mean ± SD	2.95 ± 1.89	2.85 ± 1.34	2.76 ± 1.68	0.371
eGFR <60 mL/min/1.73m^2 , %	197 (54.81)	16 (22.22)	4 (8.82)	<0.001**
S-UA (mg/dL), Mean ± SD	47.86 ± 17.90	48.63 ± 15.16	51.90 ± 20.54	0.151
S-UA >7.2 mg/dL, %	16.72	5.50	1.48	0.502
Proteinuria > 30 mg/dL	252	50	9	0.069
Blood in urine, %	24.54	6.54	0	0.313
**(B) Indicators of dehydration**				
Urinary specific gravity ≥1.0330%	45.30	31.75	21.45	0.127
Urinary pH ≤5.5 %	45.33	32.40	27.12	0.012
BUN/SCr ratio>20 %	34.77	7.02	0	<0.001**
HSP 27 mg/L (Serum)	779	703	407	<0.001**
HSP 27 mg/L (Urine)	2017	1089	553	0.11
HSP 70 mg/L(Serum)	103	0	0	0.28
HSP 70 mg/L(Urine)	801	340	215	<0.001**

** indicate significance difference at p ≤0.001;

*p Value for differences between groups: ANOVA for normally distributed continuous variables, Kruskal-Wallis for not normally distributed continuous variables, $\chi 2$ test for categorical variables.

‡Significant different from other two categories in post hoc test.

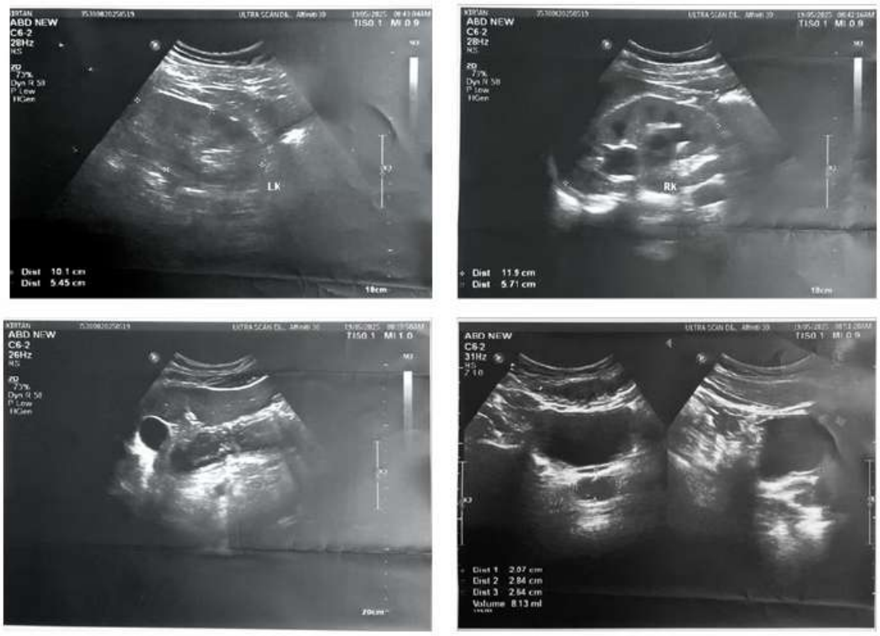

Supplementary Figure 1: Representative USG of participants with kidney stone.
